# Relationship between the Plasma Levels of Catecholamines and Return of Spontaneous Circulation in Patients with Out-of-Hospital Cardiac Arrest

**DOI:** 10.1155/2021/5324038

**Published:** 2021-07-29

**Authors:** Yumi Ichikawa, Yusuke Sawada, Jun Nakajima, Yuta Isshiki, Kazunori Fukushima, Yuto Aramaki, Kiyohiro Oshima

**Affiliations:** Department of Emergency Medicine, Gunma University Graduate School of Medicine, 3-39-22 Showa-Machi, Maebashi, Gunma 371-8511, Japan

## Abstract

**Purpose:**

The dynamic state of epinephrine (Ep) in the plasma of patients with out-of-hospital cardiac arrest (OHCA) remains unclear. The purpose of this study was to evaluate the relationship between the plasma levels of catecholamines (such as epinephrine (Ep), norepinephrine (Nep), and dopamine) and vasopressin (antidiuretic hormone (ADH)) and the acquisition of return of spontaneous circulation (ROSC) in OHCA patients.

**Methods:**

This was a prospective, observational clinical study. Patients with OHCA transferred to our hospital between July 2014 and July 2017 were enrolled. The levels of catecholamines and ADH in the plasma were measured using blood samples immediately obtained on arrival at our hospital and before the administration of Ep. Patients in whom Ep was already administered prior to obtaining blood samples were excluded. Patients were divided into two groups: with and without ROSC, that is, ROSC (+) and ROSC (−) groups, respectively. The plasma levels of these agents and the conditions of resuscitation were compared between the two groups.

**Results:**

A total of 96 patients with OHCA were analyzed. The ROSC (+) and ROSC (−) groups included 34 and 62 patients, respectively. There were no significant differences observed between the two groups in age, cause of cardiopulmonary arrest, and prehospital resuscitation time. The plasma levels of Ep and Nep were significantly lower in the ROSC (+) group than in the ROSC (−) group. However, there were no significant differences in the plasma levels of dopamine and ADH between the two groups.

**Conclusion:**

Increased levels of Ep in the plasma may not be associated with the acquisition of ROSC in patients with OHCA.

## 1. Introduction

Cardiac arrest remains a leading cause of mortality and morbidity in the United States of America and other countries worldwide [1]. Approximately, 350,000 adults in the United States of America experienced nontraumatic out-of-hospital cardiac arrest (OHCA) attended by emergency medical services personnel in 2015 [2]. Notably, approximately 10.4% of patients with OHCA survived their initial hospitalization, and 8.2% survived with good functional status. In Japan, 127,018 patients with OHCA were transferred to the hospital in 2017, and the 1-month survival rate was only 6.6% [3]. Therefore, the establishment of more effective cardiopulmonary resuscitation (CPR) is required.

Research studies regarding the role of epinephrine (Ep) as a vasopressor in CPR are continuously performed. Based on the results of numerous research studies, the 2020 International Consensus on Cardiopulmonary Resuscitation and Emergency Cardiovascular Care Science with Treatment Recommendations recommends the administration of Ep during CPR (strong recommendation and low-to-moderate certainty of the evidence) [4, 5]. At present, Ep is the only vasopressor recommended for use during CPR for patients experiencing cardiopulmonary arrest (CPA) with both shockable and nonshockable rhythms. However, the dynamic state of Ep in the plasma of patients with CPA remains unclear. In addition, the levels of catecholamines in the plasma of patients with CPA remain undetermined, and the relationship between the levels of catecholamines such as Ep, norepinephrine (Nep), and dopamine (DOA) in the plasma and the acquisition of return of spontaneous circulation (ROSC) in patients with CPA has not been understood. On the other hand, Tárnoky and Nagy [6] evaluated the relationship between the levels of catecholamines and survival in animal hemorrhagic shock and showed that the levels of catecholamines such as Ep and Nep in the plasma were significantly higher in the nonsurviving animals. They insisted that an extremely high level of catecholamines represented the stage of shock irreversibility in those animals. In addition, the toxicity of Ep to organisms has been previously reported [7–10]. There are still obscure points in the role of catecholamines in seriously critical conditions.

We postulated that the higher plasma levels of catecholamines would be associated with the higher rate of ROSC acquisition. The purpose of this study was to evaluate whether the levels of catecholamines in the plasma on arrival at the hospital affect the acquisition of ROSC in patients with OHCA.

## 2. Methods

This was a prospective, observational clinical study approved by the ethics committee of Gunma University Hospital (IRB #14-13). Written informed consent was provided by the relatives of patients with OHCA at the time of arrival at Gunma University Hospital.

Patients with OHCA transferred to Gunma University Hospital between July 2014 and July 2017 were included. CPR was required for all patients when the ambulance arrived at the scene. Prehospital care before the arrival at our hospital was performed by ambulance crews including paramedics. There is a prehospital protocol for the management of patients with OHCA in our area (Maebashi city in Gunma prefecture), and the protocol was made based on the resuscitation guidelines established in 2015 by the Japan Resuscitation Council [11]. CPR was continued after the arrival at our hospital in conformity with the same resuscitation guidelines [11].

**Figure 1:**
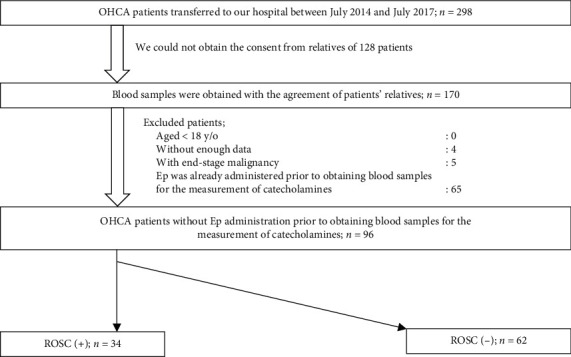
Study flow chart. Ep: epinephrine, OHCA: out-of-hospital cardiac arrest, and ROSC: return of spontaneous circulation.

The levels of catecholamines such as Ep, Nep, and DOA in the plasma were measured using blood samples obtained immediately upon arrival at the hospital and prior to the administration of Ep in the hospital. The levels of vasopressin (antidiuretic hormone (ADH)) in the plasma were also measured (we did not measure the time between the arrival at the hospital and blood collection). Blood samples were centrifuged, and the serum was stored at −80°C. The levels of catecholamines and ADH were measured using high-performance liquid chromatography and radioimmunoassay, respectively (Bio Medical Laboratories Inc., Tokyo, Japan). Patients who was already administered with Ep prior to obtaining blood samples for the measurement of catecholamines were excluded.  Figure 1 illustrates the selection criteria for patients evaluated in this study.

Successful resuscitation, that is, ROSC (+), was defined as detection of a pulse at the carotid artery, femoral artery, or radial artery under advanced CPR and subsequent maintenance of systolic pressure ≥80 mmHg for 1 h with or without the continuous administration of vasoconstrictive agents intravenously or intraosseously [12]. Patients not achieving the above criteria were defined as ROSC (−).

Patients were divided into two groups, namely, the ROSC (+) and ROSC (−) groups. The levels of the aforementioned catecholamines and ADH in the plasma, as well as the conditions of resuscitation prior to and after arrival at the hospital, were compared between the two groups. The causes of CPA were decided based on the situations at the time of patients being found, patients' comorbidities as far we were able to determine, and results of blood examinations and imaging studies such as ultrasonography and computed tomography (CT). CTs were performed in all CPA patients of this study (CTs were done after CPR in patients without ROSC). All CT images were interpreted by radiologists in our hospital. When the cause of cardiac arrest was unclear, we labelled the cause of CPA as “indeterminate.”

### 2.1. Statistical Analysis

Descriptive statistics included medians and interquartile range for continuous variables and counts, numbers, and percentages for categorical variables. Comparisons of categorical variables between the ROSC (+) and the ROSC (−) groups were performed using the chi-squared test or Fisher's exact test. Comparisons of continuous variables between the two groups were performed using the Mann‒Whitney *U* test. *P* < 0.05 denoted statistically significant difference. The IBM SPSS Statistics 26.0 software (IBM Japan, Tokyo, Japan) was used for statistical analysis.

## 3. Results

Of the 298 patients with OHCA transferred to our hospital between July 2014 and July 2017, blood samples of 170 patients were obtained with the agreement from patients' relatives. Of those 170 patients, 74 were excluded based on the exclusion criteria, and finally, 96 patients were analyzed. The ROSC (+) and the ROSC (−) groups included 34 and 62 patients, respectively (Figure 1).

Table 1 shows the comparisons between the two groups, including the conditions of resuscitation prior to and after arrival at the hospital.

There were no significant differences between the two groups in age, male/female ratio, and the rate of bystander CPR performed. There was a significant difference in the electrocardiogram waveform at the time of emergency service arriving at the scene, and the proportion of patients with ventricular fibrillation was larger in the ROSC (+) group than in the ROSC (−) group. Therefore, the frequency of prehospital defibrillation was significantly higher in the ROSC (+) group. “Prehospital resuscitation time” referred to the duration from the start of CPR to the arrival at our hospital. If bystander CPR was done, the start of bystander CPR was also the start of prehospital CPR. If bystander CPR was not performed, the start of prehospital CPR was the start of CPR by the ambulance crews. Prehospital resuscitation time was shorter in the ROSC (+) group than in the ROSC (−) group, without a significant difference. The resuscitation time after arrival at the hospital and the total resuscitation time were significantly shorter in the ROSC (+) group than in the ROSC (−) group. In addition, the dosage of Ep after arrival at the hospital was significantly lower in the ROSC (+) group.

The causes of CPA are shown in Table 2.

There was no patient in whom the cause of CPA was sepsis in the ROSC (−) group. On the other hand, the ROSC (+) group had no patient in whom the cause of CPA was acute aortic dissection, rupture of abdominal aortic aneurysm, pulmonary embolism, or drowning. As a result, there was no significant difference in the causes of CPA between the two groups.

**Figure 2:**
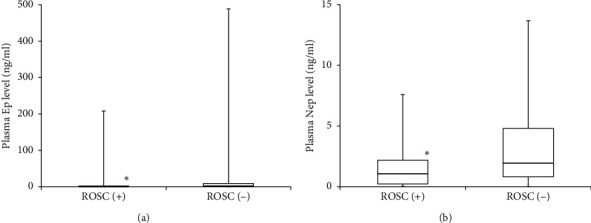
Comparisons of Ep and Nep levels in the plasma. (a) Comparison of Ep levels in the plasma. The normal levels of Ep in the plasma are ≤0.10 ng/ml (^*∗*^*P* < 0.05).(b) Comparison of Nep levels in the plasma. The normal range of Nep levels in the plasma is 0.10–0.50 ng/ml (^*∗*^*P* < 0.05). Ep: epinephrine, Nep: norepinephrine, and ROSC: return of spontaneous circulation.

The levels of Ep in the plasma (normal range: ≤0.1 ng/ml) were significantly lower in the ROSC (+) group (0.59 (0.12, 2.35) ng/ml) than in the ROSC (−) group (2.47 (0.80, 9.09) ng/ml; *P*=0.014; Figure 2(a)). The levels of Nep in the plasma (normal range: 0.10–0.50 ng/ml) were also significantly lower in the ROSC (+) group (1.06 (0.23, 2.18) ng/ml) than in the ROSC (−) group (1.94 (0.82, 4.80) ng/ml; *P*=0.027; Figure 2(b)).

**Figure 3:**
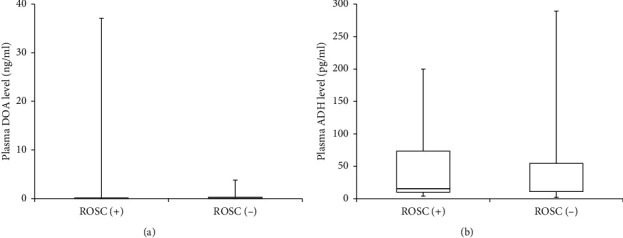
Comparisons of DOA and ADH levels in the plasma. (a) Comparison of DOA levels in the plasma. The normal level of DOA in the plasma is ≤0.03 ng/ml. (b) Comparison of ADH levels in the plasma. The normal level of ADH in the plasma is ≤4.2 pg/ml. DOA: dopamine and ADH: antidiuretic hormone (vasopressin).

There were no significant differences in the plasma levels of DOA (normal range: ≤0.03 ng/ml) and ADH (normal range: ≤4.2 pg/ml) between the two groups (DOA: 0.07 (0.01, 0.16) ng/ml and 0.07 (0.03, 0.27) ng/ml, *P*=0.471; ADH: 15.50 (10.00, 73.55) pg/ml and 11.00 (11.00, 54.40) pg/ml, *P*=0.842, in the ROSC (+) and ROSC (−) groups, respectively; Figures 3(a) and 3(b)).

## 4. Discussion

This study showed that the levels of catecholamines, such as Ep, Nep, and DOA, and ADH measured in blood samples obtained before the administration of Ep in patients with OHCA exceeded the normal ranges in both ROSC (+) and ROSC (−) groups. In addition, the levels of Ep and Nep in the plasma prior to the administration of Ep were significantly lower in the ROSC (+) group than in the ROSC (−) group. There was no significant difference in the duration of prehospital CPR, and prehospital defibrillation was not performed in any patient of the ROSC (−) group. In the ROSC (+) group, the dosages of Ep and resuscitation time after arrival at the hospital were significantly lower and shorter, respectively. These results coincide with the findings of our previous report [12]. This suggests that the levels of endogenous Ep and Nep in the plasma before the administration of Ep are markedly increased in patients with OHCA, and those levels may not be related to the length of resuscitation time and the frequency of defibrillation. In addition, it is suspected that the increase in the plasma levels of Ep by additional administration does not contribute to achieving ROSC in patients with OHCA.

At present, Ep is the only vasopressor recommended for use during CPR in patients experiencing CPA with both shockable and nonshockable rhythms [4, 5]. Nevertheless, concerns have been expressed about Ep increasing the number of survivors with unfavorable neurological outcomes. The PARAMEDIC2 trial showed that the use of Ep resulted in a significantly higher rate of 30-day survival versus placebo; however, there was no significant between-group difference in the rate of a favorable neurologic outcome between the use of Ep and placebo [13]. Kempton et al. performed a systematic review and meta-analysis of Ep versus placebo in patients with OHCA. They reported that the use of Ep was not associated with a significant difference in survival to hospital discharge, neurological outcomes, or 3-month survival [14]. Aves et al. also performed an updated systematic review and meta-analysis of clinical trials evaluating Ep for the resuscitation of adults with OHCA. They reported that administration of the standard dose of Ep improved overall survival but not neurologic outcomes in patients with OHCA versus placebo [15]. Those studies showed that the administration of Ep does not lead to satisfactory outcomes in the long-term prognosis of patients with OHCA.

The toxicity of Ep to organisms has been previously reported. Berg et al. showed that high-dose Ep resulted in severe tachycardia, hypertension, and a higher mortality rate immediately after resuscitation [7]. Evidence points to Ep as a precipitating factor of takotsubo syndrome, which is recognized as acute and severe but reversible heart failure [8–10]. In addition, Lu et al. reported that continuous intravenous administration of Ep decreased stroke volume in animal models [16]. Nonetheless, Ristagno et al. reported that the administration of Ep decreased the cerebral capillary blood flow [17–19] and cerebral cortical oxygen tension [19] in animal models with CPA. Recently, Mavroudis et al. showed that Ep increased cerebral blood flow and cerebral tissue oxygenation; however, these effects waned following the third dose [20]. Collectively, the results of previous reports and the present study suggested that the elevation in the plasma levels of Ep induced by the exogenous administration of Ep adversely affects the cerebral circulation and neurological prognosis in patients with CPA.

DOA is a precursor of Nep and converts to Nep through DOA *β*-hydroxylase [21]. In this study, there was no significant difference in the levels of DOA in the plasma between the two groups. Our results suggest that the exogenous administration of Ep does not influence the plasma levels of DOA.

There are three randomized controlled trials involving more than 1,500 patients with OHCA comparing ADH versus Ep; however, all those studies were published before 2010 [22–24]. The combined results of those studies did not show benefits of ADH compared with Ep across all outcomes and initial rhythms. The 2020 International Consensus on CPR and Emergency Cardiovascular Care Science with Treatment Recommendations suggests against the administration of ADH in place of Ep during CPR with weak recommendation and very low-certainty evidence [4, 5]. In this study, the plasma levels of ADH exceeded the normal range and were similar between the two groups. It is suggested that the increase in the plasma levels of Ep does not influence the plasma levels of ADH in patients with OHCA.

There were some limitations in the present study. This was a prospective monocentric study, involving a small number of patients. The cause of CPA was complex and included both endogenous and exogenous etiologies. The disproportion of blood distribution by CPR may indicate the levels of Ep in the plasma. In this study, we evaluated only the levels of catecholamines and ADH in the plasma before the administration of Ep, and the influence of exogenous Ep was not studied at all. While our research does not necessarily deny the need to administer Ep in CPA patients, our data suggests that we may need to reconsider the method of administering Ep including its dosage and interval. We strongly think that the dynamics of catecholamines in CPA patients should be investigated more in-depth in view of our current provisional results.

In conclusion, increased levels of Ep in the plasma may not be associated with the acquisition of ROSC in patients with OHCA.

## Figures and Tables

**Table 1:** Comparisons between the two groups.

	ROSC (+) (*n* = 34)	ROSC (–) (*n* = 62)	*P* value
Age (years)	80.5 (68.3, 85.5)	82.0 (72.3, 87.0)	0.642
Male/female ratio	21/13	31/31	0.269
Bystander CPR	20 (58.8%)	28 (45.6%)	0.200
ECG at arrival of emergency service			<0.001^*∗*^
VF	8	0	
PEA	14	13	
Asystole	12	49	
Prehospital resuscitation time (min)	16.0 (12.3, 20.8)	18.0 (15.0, 23.0)	0.053
Frequency of prehospital defibrillation	0 (0, 1)	0 (0, 0)	<0.001^*∗*^
Resuscitation time after arrival at the hospital (min)	8.0 (0.8, 9.0)	23.5 (15.3, 33.0)	<0.001^*∗*^
Dosage of Ep after arrival at the hospital (mg)	1.0 (0, 2.0)	4.0 (3.0, 5.0)	<0.001^*∗*^
Total resuscitation time (min)	25.5 (19.3, 30.0)	44.0 (33.0, 51.0)	<0.001

*Note.* CPR: cardiopulmonary resuscitation, ECG: electrocardiogram, Ep: epinephrine, PEA: pulseless electrical activity, ROSC: return of spontaneous circulation, and VF: ventricular fibrillation. Data are shown as number or median (*Q*1, *Q*3). ^*∗*^*P* < 0.05.

**Table 2:** Comparison of the causes of CPA between the two groups.

	ROSC (+) (*n* = 34)	ROSC (−) (*n* = 62)	*P* value
Causes of CPA			0.102
Indeterminate	14	34	
Respiratory	7	4	
Choking	7	9	
Intracranial hemorrhage	3	1	
Sepsis	1	0	
Trauma	1	2	
Gastrointestinal bleeding	1	1	
Acute aortic dissection	0	7	
AAA rupture	0	2	
Pulmonary embolism	0	1	
Drowning	0	1	

*Note.* AAA: abdominal aortic aneurysm, CPA: cardiopulmonary arrest, and ROSC: return of spontaneous circulation.

## Data Availability

The data in this manuscript were obtained from OHCA patients transferred to Gunma University Hospital between July 2014 and July 2017. Data used in this manuscript can be obtained from the corresponding author upon request.
